# Comparative Study on Multimodal Imaging Applications in Dementia with Lewy Bodies: From Imaging Features to Clinical Practice Implications

**DOI:** 10.3390/brainsci15121264

**Published:** 2025-11-25

**Authors:** Qijun Li, Zhaoxia Huang, Junshan Wang, Menglin Liang, Chenhao Jia, Jing Yuan, Ruixue Cui

**Affiliations:** 1 Department of Nuclear Medicine, Peking Union Medical College Hospital, Chinese Academy of Medical Sciences and Peking Union Medical College, No. 1 Shuaifuyuan, Wangfujing, Dongcheng District, Beijing 100730, China; liqijun210@163.com (Q.L.);; 2 Beijing Key Laboratory of Molecular Targeted Diagnosis and Therapy in Nuclear Medicine, Beijing 100730, China; 3 Department of Neurology, Peking Union Medical College Hospital, Chinese Academy of Medical Sciences and Peking Union Medical College, No. 1 Shuaifuyuan, Wangfujing, Dongcheng District, Beijing 100730, China

**Keywords:** dementia with Lewy body, ^18^F-Fluorodeoxyglucose, positron emission tomography/computed tomography

## Abstract

**Objective**: To explore the value of multimodal molecular imaging in diagnosing and differentiating dementia with Lewy bodies (DLB). **Methods:** A retrospective analysis was conducted on clinical data and multimodal molecular imaging of 40 probable DLB patients treated at Peking Union Medical College Hospital (August 2017–December 2024). All 40 had ^18^F-FDG PET/CT; 15 had ^131^I-MIBG imaging; 11 had ^18^F-FP-CIT PET/CT. A total of 12 patients with poor cognition or atypical ^18^F-FDG PET/CT underwent ^18^F-AV45 PET/CT (2 also had ^18^F-PM-PBB3 imaging). A sex- and age-matched control group (cognitively normal, same-period health checkup ^18^F-FDG PET/CT) was included. ^18^F-FDG PET/CT images were visually and semi-quantitatively analyzed (ROI, SPM). ^18^F-AV45 PET/CT was assessed both visually and semi-quantitatively; ^131^I-MIBG imaging and ^18^F-FP-CIT PET/CT were visually evaluated. **Results:** The 40 DLB patients (29 males, 11 females; mean age 72 years) had distinct initial symptoms: 8 (20%) presented with cognitive decline as the first symptom, 23 (57.5%) with parkinsonian symptoms as the first symptom, and 9 (22.5%) with both symptoms occurring simultaneously. Mean intervals: 16.25 months from initial cognitive decline to parkinsonian symptoms, and 24.43 months from initial parkinsonian symptoms to cognitive decline. All had parkinsonian symptoms and cognitive impairment; 38 (95%) had visual hallucinations; and 26 (65%) had REM sleep behavior disorder. ^18^F-FDG PET/CT: 30(75%) showed typical occipital hypometabolism and posterior cingulate island sign; 10 (25%) had atypical findings. ^131^I-MIBG (15/15, 100%): cardiac sympathetic denervation. ^18^F-FP-CIT (10/11, 90.9%): basal ganglia dopaminergic damage. ^18^F-AV45 (9/12, 81.8%): positive. Semi-quantitative ^18^F-FDG analysis revealed parietal, occipital, and lateral temporal hypometabolism in DLB (left more severe than right). **Conclusions:** Dementia with Lewy bodies (DLB) presents with pre-onset parkinsonism and cognitive impairment, plus high rates of visual hallucinations and sleep disorders. Key imaging features—occipital hypometabolism/island sign on ^18^F-FDG PET/CT, cardiac sympathetic denervation on ^131^I-MIBG, and basal ganglia dopaminergic damage on ^18^F-FP-CIT—aid DLB diagnosis. ^18^F-AV45 PET/CT detects Aβ pathology in severely cognitively impaired patients, suggesting these DLB patients may have underlying AD pathology beyond DLB.

## 1. Introduction

Dementia with Lewy bodies (DLB) is a common neurodegenerative dementia, ranking second in incidence among neurodegenerative dementias, only after Alzheimer’s disease (AD) [1,2,3,4]. DLB presents complex clinical manifestations, including cognitive, motor, neuropsychiatric, and sleep disorders. Its core clinical features (the “four main signs”) are fluctuating cognitive impairment, parkinsonian motor symptoms, visual hallucinations, and REM sleep behavior disorder. The onset is insidious, progression is rapid, and prognosis is poor.

The clinical manifestations of Dementia with Lewy Bodies (DLB) lie between those of Alzheimer’s Disease (AD) and Parkinson’s Disease (PD), and overlap with the clinical manifestations of various neurological and psychiatric diseases, which easily leads to confusion.

Fluctuating cognitive impairment is prone to be confused with the progressive cognitive impairment of AD [5,6]. Parkinsonian symptoms are also seen in PD; in addition, obvious psychiatric symptoms may appear in the early stage of DLB [7], which is easily misdiagnosed as psychosis. However, DLB patients may be extremely sensitive to antipsychotic drugs, especially traditional antipsychotics, which can easily cause serious adverse reactions, possibly aggravating DLB symptoms and even endangering life [8,9].

DLB may also coexist with AD [10,11,12,13,14], making the diagnosis more complicated. Accurate diagnosis of DLB is the premise for formulating individualized treatment plans. It is particularly important to correctly identify and diagnose DLB in its early stage. Multimodal molecular imaging plays an extremely important role in the diagnosis of DLB.

The core pathological change in DLB is the presence of intracytoplasmic inclusions (Lewy bodies) formed by aggregated α-synuclein (α-Syn) in cortical neurons [15,16]. Lewy bodies primarily involve the cortex (e.g., temporal lobe, parietal lobe, prefrontal lobe), contributing to fluctuating cognitive impairment, limbic system (e.g., amygdala, contributing to neuropsychiatric symptoms such as visual hallucinations), and brainstem nuclei (contributing to parkinsonian motor symptoms and sleep disorders). Pathological examination of brain tissue—specifically, post-mortem or biopsy detection of extensive Lewy bodies and/or Lewy neurites—is the gold standard for confirming DLB, making ante-mortem pathological diagnosis challenging. Moreover, Lewy bodies are not unique to DLB; they can also occur in other neurodegenerative diseases such as Parkinson’s disease (PD) [17] and multiple system atrophy (MSA) [18,19], complicating diagnosis.

Diagnostic criteria for DLB mainly include the fourth consensus report of the International DLB Consortium, Diagnosis and Management of Dementia with Lewy Bodies (2017) [10,20], and the Chinese Guidelines for the Diagnosis and Treatment of Dementia with Lewy Bodies (2021) issued by the Neurodegenerative Disease Professional Committee of the Chinese Microcirculation Society [21]. These guidelines classify clinical diagnoses of DLB into “probable DLB” and “possible DLB.” Probable DLB is diagnosed when two or more core clinical features are present (with or without positive supportive biomarkers), or when one core clinical feature is present with one or more positive supportive biomarkers. A diagnosis of probable DLB cannot be based solely on positive biomarkers. Biomarkers are categorized by evidence strength: supportive biomarkers and indicative biomarkers. Indicative biomarkers include multiple imaging and nuclear medicine tests, such as reduced basal ganglia dopamine transporter uptake on PET/CT, and cardiac sympathetic denervation on MIBG scintigraphy. Additional supportive biomarkers include relatively preserved medial temporal lobe structure on CT/MRI [22], and generalized hypoperfusion or hypometabolism on SPECT/PET perfusion/metabolism scans [3], with ^18^F-FDG PET/CT showing decreased occipital lobe activity with or without the cingulate island sign [23]. These criteria highlight the pivotal role of nuclear medicine in DLB diagnosis.

This study summarizes the clinical, imaging, and nuclear medicine findings of patients with probable DLB diagnosed in our hospital, aiming to explore the clinical manifestations of DLB and the value of multimodal molecular imaging in assisting DLB diagnosis and identifying comorbidities.

## 2. Subjects and Methods

Study subjects: Forty patients with probable DLB diagnosed in the Department of Neurology, Peking Union Medical College Hospital, Chinese Academy of Medical Sciences, from August 2017 to December 2024 (hereinafter referred to as the DLB group). The control group consisted of individuals who underwent ^18^F-FDG PET/CT for health checkups in the Department of Nuclear Medicine, Peking Union Medical College Hospital, during the same period, with no history of neurological or psychiatric diseases.

This study was conducted in accordance with the principles of the Declaration of Helsinki and approved by the hospital ethics committee.

### 2.1. Methods

#### 2.1.1. Clinical and Laboratory Data Collection

Clinical data included medical history, physical examination, parkinsonian symptoms, signs, and abnormal neuropsychiatric symptoms. Cognitive function was assessed using Mini-Mental State Examination (MMSE) [24] and Montreal Cognitive Assessment (MoCA) [25].

Laboratory tests included Apo E genotyping [26], blood routine, liver function, and renal function.

#### 2.1.2. Preparation, Injection, Image Acquisition, and Precautions for PET/CT and SPECT Tracers

PET tracers (^18^F-FDG, ^18^F-FP-CIT [27], ^18^F-AV45 [28], and ^18^F-PM-PBB3 [29]) were synthesized in-house by the Department of Nuclear Medicine, Peking Union Medical College Hospital, using an ALL IN ONE synthesis module, with radiochemical purity > 95%. ^18^F-AV45 is a PET tracer for β-amyloid (Aβ), ^18^F-FP-CIT for dopamine transporters (DAT), and ^18^F-PM-PBB3 for tau protein.

All tracers were administered intravenously. For ^18^F-FDG PET/CT, all subjects (DLB group and control group) fasted for at least 6 h before injection, with blood glucose < 11.1 mmol/L. The injection doses were: 5.55 MBq/kg for ^18^F-FDG, 5.55 MBq/kg for ^18^F-AV45, 5.55 MBq/kg for ^18^F-PM-PBB3, and 3.7 MBq/kg for ^18^F-FP-CIT. Patients discontinued anti-parkinsonian drugs 12 h before ^18^F-FP-CIT PET/CT.

After ^18^F-FDG injection, patients rested in a quiet, dark environment for 40–60 min before brain imaging using a PET/CT scanner (Polestar m680, Sinowhole Co., Ltd., Nanjing, China). Low-dose cranial CT (120 kV, 150 mA, 10 s) was performed first for attenuation correction, followed by PET imaging for 10 min. For ^18^F-AV45, ^18^F-FP-CIT, and ^18^F-PM-PBB3 PET/CT, low-dose cranial CT (120 kV, 150 mA, 10 s) was performed for attenuation correction, followed by PET imaging for 20 min. Brain PET images were reconstructed using three-dimensional (3D) ordered subsets expectation maximization (OSEM).

The SPECT tracer ^131^I-MIBG was prepared by Atom High-Tech Co., Ltd. (Beijing, China) and administered intravenously. Drugs affecting ^131^I-MIBG uptake (e.g., certain antihypertensives, antidepressants) were discontinued before the examination, with the duration varying by drug type (usually several days to weeks). Patients received 111 MBq of ^131^I-MIBG. Early (15 min post-injection) and delayed (4 h post-injection) anterior–posterior static planar images were acquired, covering the chest and upper abdomen (entire liver), with a matrix size of 256 × 256. Patients took iodine supplements starting 3 days before the examination to block thyroid uptake.

#### 2.1.3. Analysis of ^18^F-FDG PET/CT Images

(1) Visual analysis: Two experienced nuclear medicine physicians from Peking Union Medical College Hospital independently interpreted ^18^F-FDG PET/CT images without reference to clinical data, assessing ^18^F-FDG metabolism in cortical and subcortical nuclei, with a focus on the occipital lobe and posterior cingulate gyrus, to diagnose and differentiate diseases. Discrepancies were resolved by a senior physician. DLB patients with atypical ^18^F-FDG metabolism patterns (e.g., bilateral medial temporal hypometabolism, posterior cingulate hypometabolism, or global hypometabolism) were classified into the “atypical DLB subgroup” (*n* = 10).
(2) Voxel-wise statistical analysis of ^18^F-FDG PET/CT brain functional imaging data was performed using Statistical Parametric Mapping 12 (SPM12), including preprocessing and statistical analysis. Preprocessing steps were:
  ① Spatial normalization: To eliminate inter-individual differences, all data were normalized to the Montreal Neurological Institute (MNI) space using the DARTEL algorithm and resampled to a resolution of 2 × 2 × 2 mm^3^.
  ② Gaussian smoothing: Normalized data were spatially smoothed with an 8 mm full-width at half-maximum Gaussian kernel to approximate a normal distribution.
  ③ Calculation of standardized uptake value ratio (SUVR): To correct for variations in injection dose, imaging time, and basal metabolic rate, the cerebellar cortex was used as the reference region, and SUVR was calculated voxel-wise as the ratio of each voxel’s uptake to the mean uptake in the reference region.

Voxel-wise two-sample t-tests were performed on preprocessed SUVR images to compare the entire DLB group (*n* = 40), the atypical DLB subgroup (*n* = 10), and the control group (*n* = 40). The significance threshold for inter-group differences was *p* < 0.05 (FWE-corrected), with a cluster level of Ke > 50.

Brain regions with statistically significant differences (*p* < 0.05) were identified as having abnormal metabolism. These regions were visualized on axial T1-weighted (T1WI) standard templates using xjView software (version 9.6).

#### 2.1.4. Analysis of ^18^F-AV45, ^18^F-FP-CIT, and ^18^F-PM-PBB3 PET/CT Images

Visual interpretation and semi-quantitative analysis were performed for these PET/CT images. Two experienced nuclear medicine physicians interpreted the images without reference to clinical data: ^18^F-AV45 and ^18^F-PM-PBB3 PET/CT focused on the cerebral cortex, while ^18^F-FP-CIT PET/CT focused on the basal ganglia. For ^18^F-AV45 PET/CT, semi-quantitative analysis included calculation of centiloid (CL) values [30] using quantitative software SPM12.

#### 2.1.5. Analysis of ^131^I-MIBG Scintigraphy

Visual interpretation of SPECT images classified cardiac sympathetic innervation as either “normal” or “impaired.” Using the mediastinum as the background, clear tracer uptake in the cardiac region indicated normal cardiac sympathetic innervation, while blurred uptake indicated impairment.

#### 2.1.6. Statistical Analysis

Statistical analysis was performed using Statistical Product and Service Solutions (SPSS) 26.0 software. Normally distributed quantitative data (e.g., age, SUVR, MMSE scores) were expressed as mean ± standard deviation. Inter-group comparisons were performed using independent samples *t*-tests, with statistical significance set at *p* < 0.05. Voxel-wise inter-group comparisons were analyzed using two-sample *t*-tests with FWE correction, and significance was set at *p* < 0.05.

## 3. Results

### 3.1. Clinical and Laboratory Data

#### 3.1.1. General Data of the DLB Group and Control Group

The DLB group included 40 patients (29 males, 11 females) with a mean age of 72.1 ± 6.9 years and a mean MMSE score of 18.1 ± 5.5. The control group included 40 age- and sex-matched individuals with a mean age of 72.1 ± 8.0 years. There were no significant differences in age or sex between the two groups (*p* > 0.05).

Laboratory data showed that 8 patients had the Apo E3/4 genotype, and the remaining had Apo E3/3 or 2/3 genotypes. General data of all subjects are shown in Table 1.

#### 3.1.2. Analysis of Clinical Manifestations

In the DLB group, 8 patients (20%) presented with cognitive decline as the initial symptom, and 23 patients (57.5%) presented with parkinsonian symptoms as the initial symptom: 9 patients (22.5%) had concurrent onset of both symptoms. The average interval from the onset of initial cognitive decline to the development of parkinsonian symptoms was 16.25 months, and the average interval from the onset of initial parkinsonian symptoms to the development of cognitive decline was 24.43 months.

All 40 patients (100%) had parkinsonian symptoms (mainly bradykinesia and tremor) and cognitive impairment; 38 (95%) had visual hallucinations, and 26 (65%) had REM sleep behavior disorder.

### 3.2. Imaging Results

#### 3.2.1. Visual Analysis of ^18^F-FDG PET/CT

**Figure 1:**
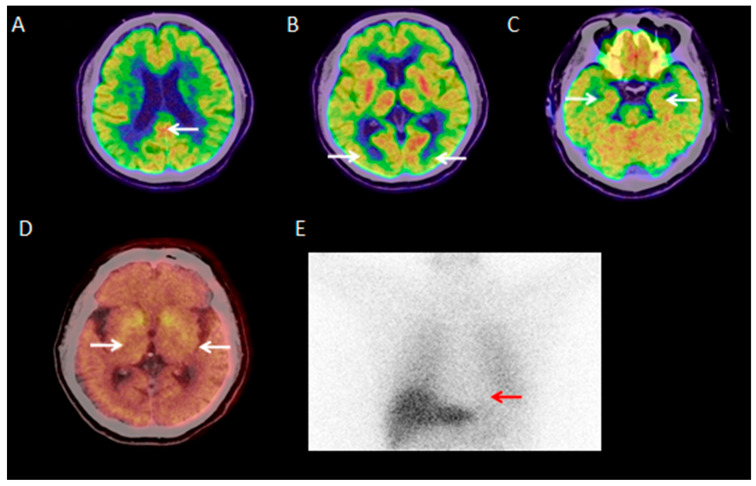
A 69-year-old male patient with a 5-year history of bradykinesia and unsteady gait, and visual hallucinations (reporting “fighting little devils” at night and perceiving hats/plastic bags as skulls). ^18^F-FDG PET/CT shows preserved posterior cingulate metabolism (posterior cingulate island sign), bilateral occipital hypometabolism, and preserved bilateral medial temporal metabolism ((**A**–**C**); white arrows). ^18^F-FP-CIT PET/CT shows reduced uptake in the bilateral putamen tails ((**D**); white arrow). ^131^I-MIBG scintigraphy indicates cardiac sympathetic denervation ((**E**); red arrow).

**Figure 2:**
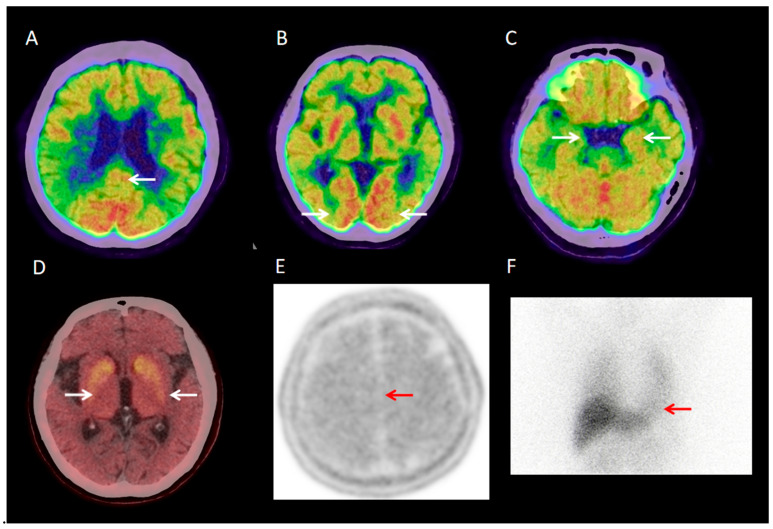
A 71-year-old female patient with 3-year fluctuating cognitive impairment and visual hallucinations. ^18^F-FDG PET/CT shows preserved posterior cingulate metabolism (posterior cingulate island sign), bilateral parieto-occipital hypometabolism, and medial temporal hypometabolism ((**A**–**C**); white arrows). ^18^F-FP-CIT PET/CT shows reduced uptake in the bilateral putamen tails ((**D**); white arrow). ^18^F-AV45 PET shows diffuse cortical Aβ deposition ((**E**); red arrow). ^131^I-MIBG scintigraphy indicates cardiac sympathetic denervation ((**F**); red arrow).

Visual analysis showed that 30 of 40 DLB patients (30/40, 75%) had typical ^18^F-FDG PET/CT findings: occipital hypometabolism and a posterior cingulate island sign (Figure 1A–C). The remaining 10 patients (10/40, 25%) had atypical findings, such as generalized global hypometabolism or significant medial temporal hypometabolism (Figure 2A–C).

#### 3.2.2. ROI-Based Semi-Quantitative Analysis

Compared with the control group (*n* = 40), the DLB group (*n* = 40) showed ^18^F-FDG hypometabolism in the parietal lobe, occipital lobe, and lateral temporal lobe on PET, with relatively preserved hippocampal metabolism. ROI-based semi-quantitative analysis confirmed the metabolic pattern characterized by hypometabolism in the occipital lobe (right/left calcarine cortex, right/left cuneus, right/left superior/middle/inferior occipital gyri), parietal lobe (right/left precuneus, right/left angular gyrus), lateral temporal lobe (right/left middle/inferior temporal gyri), and posterior cingulate gyrus, with relatively preserved bilateral hippocampal metabolism and relatively increased bilateral amygdalar metabolism (Table 2).

#### 3.2.3. Voxel-Based Semi-Quantitative Analysis

**Figure 3:**
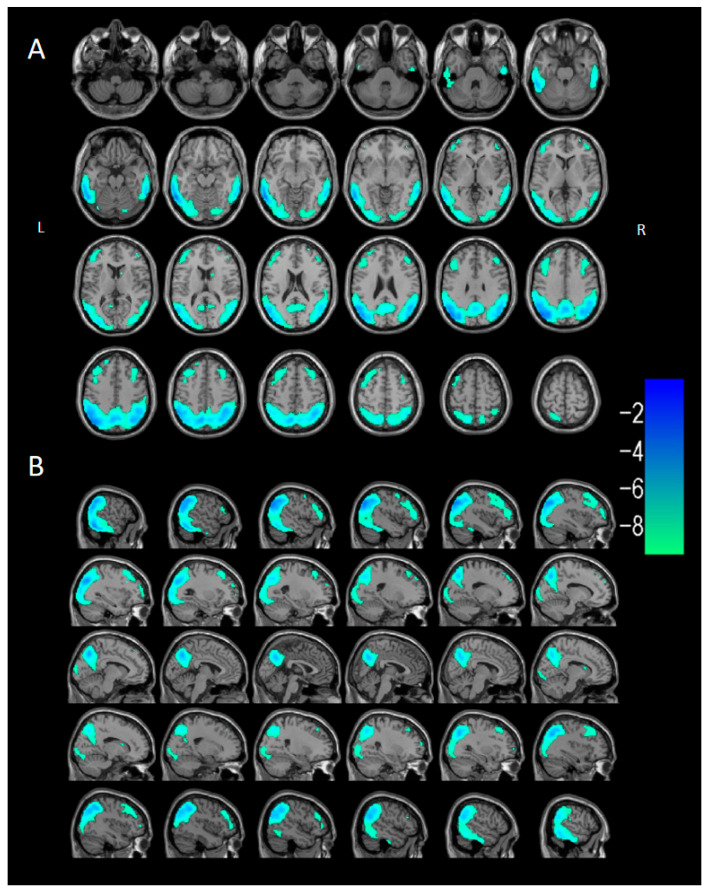
Two-sample *t*-test of voxel-wise differences between the DLB group (*n* = 40) and control group (*n* = 40). SPM voxel-based semi-quantitative analysis of brain metabolism was visualized using xjView software on a standard T1WI brain template. (**A**) Serial axial sections (5 mm intervals) showing hypometabolism in the bilateral frontal, parietal, occipital, and lateral temporal cortices. (**B**) Serial sagittal sections (4 mm intervals) showing hypometabolism in the bilateral frontal, parietal, occipital, and lateral temporal cortices, with no hypometabolism in the posterior cingulate gyrus. Cool colors (blue, green)indicate hypometabolic region.

Compared with the control group (*n* = 40), the DLB group (*n* = 40) showed ^18^F-FDG hypometabolism in the bilateral occipital lobe (middle/inferior/superior occipital gyri, cuneus, calcarine cortex, lingual gyrus), bilateral parietal lobe (inferior/superior parietal lobules, angular gyrus, supramarginal gyrus, precuneus), lateral temporal lobe (bilateral inferior/middle/superior temporal gyri, left fusiform gyrus), and frontal lobe (bilateral middle/inferior frontal gyri, left superior frontal gyrus) on PET (Figure 3 and Table 3), with few hypometabolic voxels in the posterior cingulate cortex (<100). Voxel-based semi-quantitative analysis confirmed the metabolic pattern of parietal, occipital, and lateral temporal hypometabolism observed visually. Additionally, left-sided hypometabolism was more extensive than right-sided in DLB patients.

**Figure 4:**
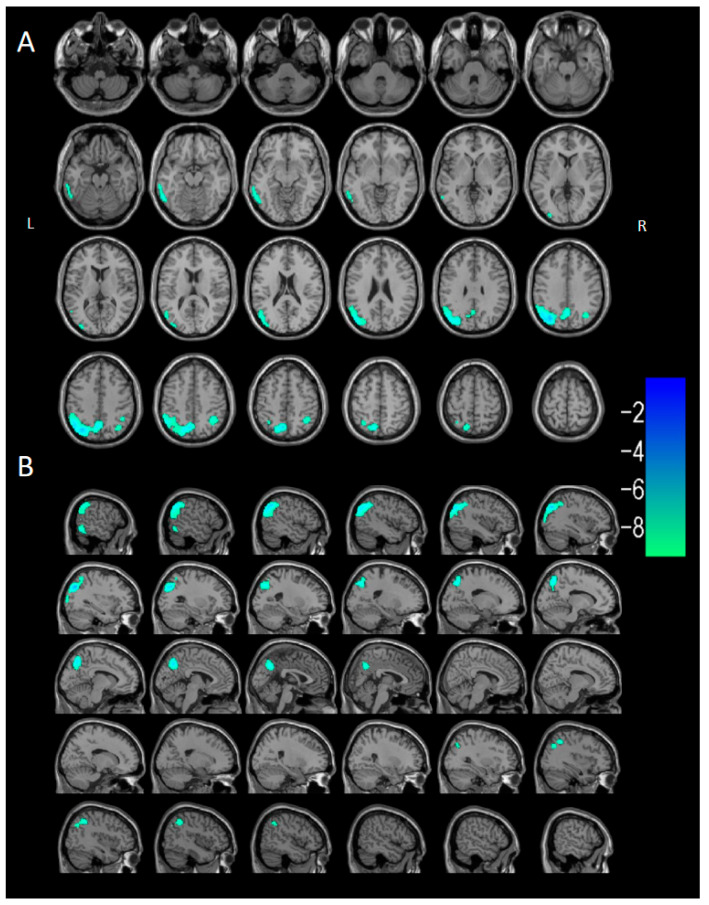
Two-sample *t*-test of voxel-wise differences between the atypical DLB subgroup (*n* = 10) and control group (*n* = 40). SPM voxel-based semi-quantitative analysis of brain metabolism was visualized using xjView software on a standard T1WI brain template. (**A**) Serial axial sections (5 mm intervals) showing hypometabolism in the bilateral frontal, parietal, occipital, and lateral temporal cortices. (**B**) Serial sagittal sections (4 mm intervals) showing hypometabolism in the left temporal lobe (middle/inferior temporal gyri), left parietal lobe (angular gyrus, precuneus), right parietal lobe (inferior parietal angular gyrus), and left occipital lobe, with no hypometabolism in the posterior cingulate gyrus. Cool colors (blue, green) indicate hypometabolic regions.

Voxel-based semi-quantitative analysis comparing the atypical DLB subgroup (*n* = 10) with the control group (*n* = 40) showed hypometabolism in the left temporal lobe (middle/inferior temporal gyri), left parietal lobe (angular gyrus, precuneus), right parietal lobe (inferior parietal angular gyrus), and left occipital lobe in the atypical DLB subgroup (Figure 4).

#### 3.2.4. Visual Analysis of ^18^F-AV45 PET/CT

**Figure 5:**
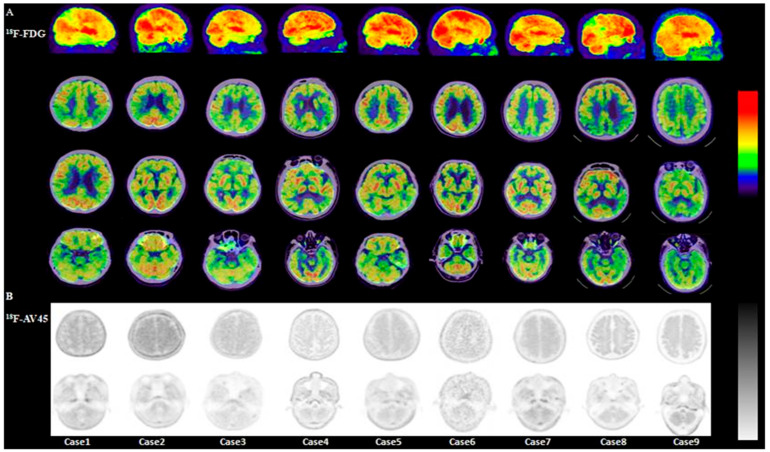
^18^F-AV45 PET imaging in 12 patients with atypical ^18^F-FDG PET/CT findings or DLB symptoms showed diffuse cortical Aβ deposition in 9. ^18^F-FDG PET/CT findings in these patients were: Case 1: posterior cingulate hypometabolism, medial temporal hypometabolism; Cases 2–7: medial temporal hypometabolism; Case 8: medial temporal and parietal hypometabolism; Case 9: global hypometabolism.

Twelve patients—2 with atypical DLB symptoms (more severe cognitive decline than parkinsonian symptoms, indistinct visual hallucinations) and 10 with atypical ^18^F-FDG PET/CT findings—underwent ^18^F-AV45 PET/CT. Among them, 9 (9/12, 81.8%) showed diffuse increased radiotracer uptake in the cerebral cortex, with uptake intensity approaching white matter, including “kiss sign” and “cypress tree sign,” indicating positive ^18^F-AV45 PET/CT results (Figure 5).

Visual analysis of ^18^F-AV45 PET in these 9 patients showed: Cases 1, 3, 4, 5, 6, and 7 had diffuse cortical Aβ deposition, with similar deposition in the cerebellar cortex. Cases 2, 8, and 9 had diffuse cortical Aβ deposition, with less cerebellar deposition than cortical.

#### 3.2.5. Semi-Quantitative Analysis of ^18^F-AV45 PET/CT

Semi-quantitative analysis of ^18^F-AV45 PET included cerebral SUV, cerebellar SUV, cerebral SUVR, and CL values. Results for cerebral SUV, cerebellar SUV, and CL values are shown in Table 4.

#### 3.2.6. Visual Analysis of ^18^F-FP-CIT PET/CT

**Figure 6:**
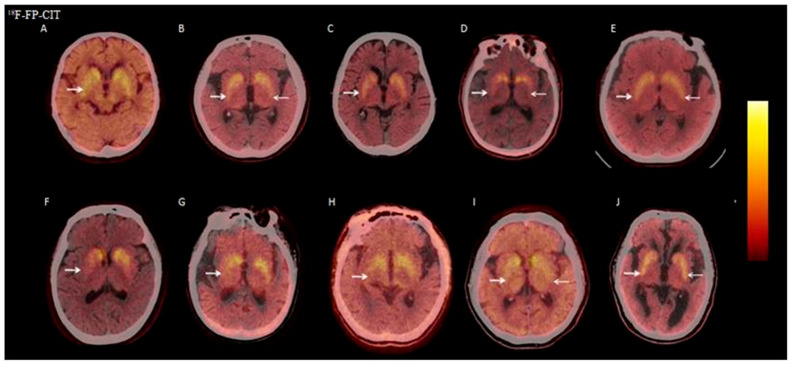
Eleven DLB patients underwent ^18^F-FP-CIT PET/CT, with 10 (10/11, 90.9%) showing reduced basal ganglia dopamine transporter distribution. (**A**,**C**,**F**–**H**) show asymmetric bilateral reduction, more severe on the right. (**B**,**D**,**E**,**I**,**J**) show symmetric bilateral reduction.

Eleven patients in the DLB group underwent ^18^F-FP-CIT PET/CT, with 10 (10/11, 90.9%) showing dopaminergic neuron damage in the basal ganglia. Among these 10 patients, 5 had symmetric bilateral reduction, and 5 had asymmetric bilateral reduction (Figure 6).

#### 3.2.7. Visual Analysis of ^131^I-MIBG SPECT

**Figure 7:**
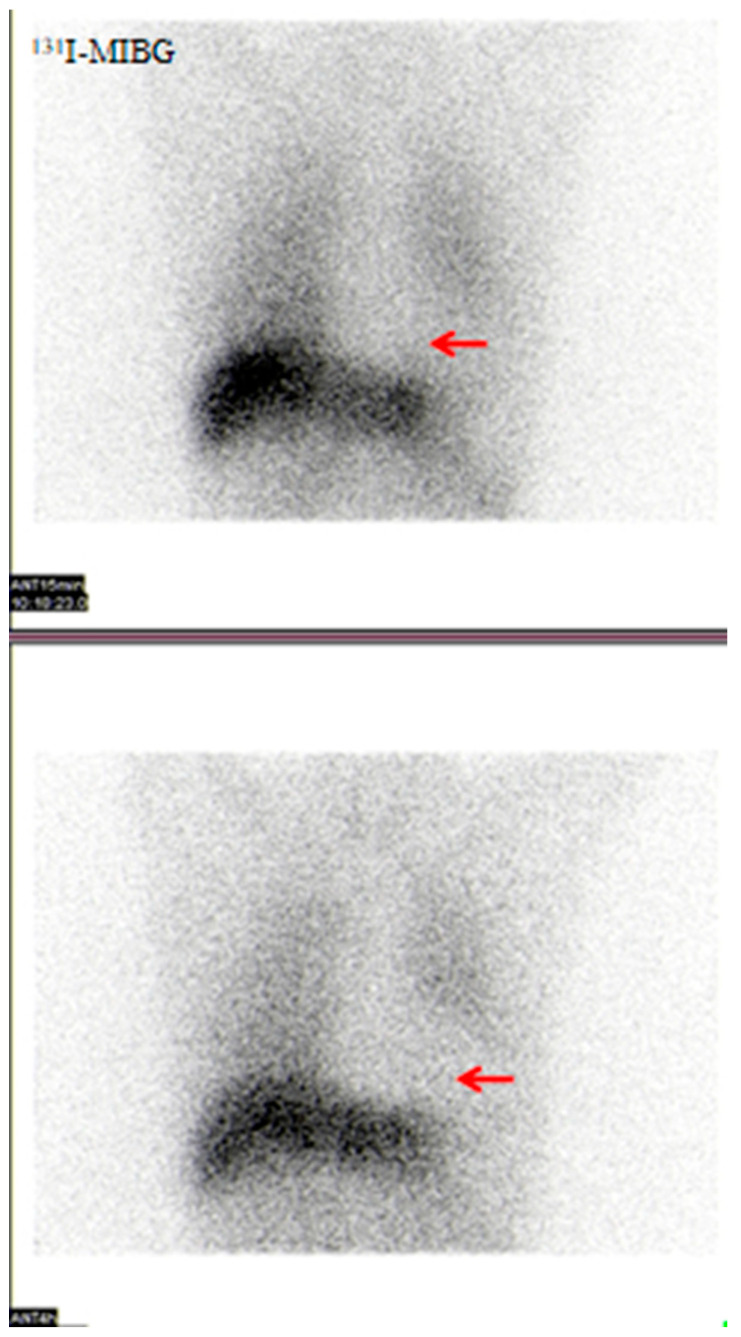
A 75-year-old male patient with 5-year fluctuating cognitive impairment and 4-year visual hallucinations. Chest imaging 15 min and 4 h after intravenous ^131^I-MIBG injection showed no significant tracer accumulation in the cardiac region (red arrow) with blurred cardiac contours, indicating cardiac sympathetic denervation.

Fifteen patients in the DLB group underwent ^131^I-MIBG scintigraphy. All 15 (15/15, 100%) showed no significant tracer accumulation in the cardiac region with blurred cardiac contours, indicating cardiac sympathetic denervation. Figure 7 shows typical cardiac sympathetic denervation in one patient.

#### 3.2.8. Visual Analysis of ^18^F-PM-PBB3 PET/CT

**Figure 8:**
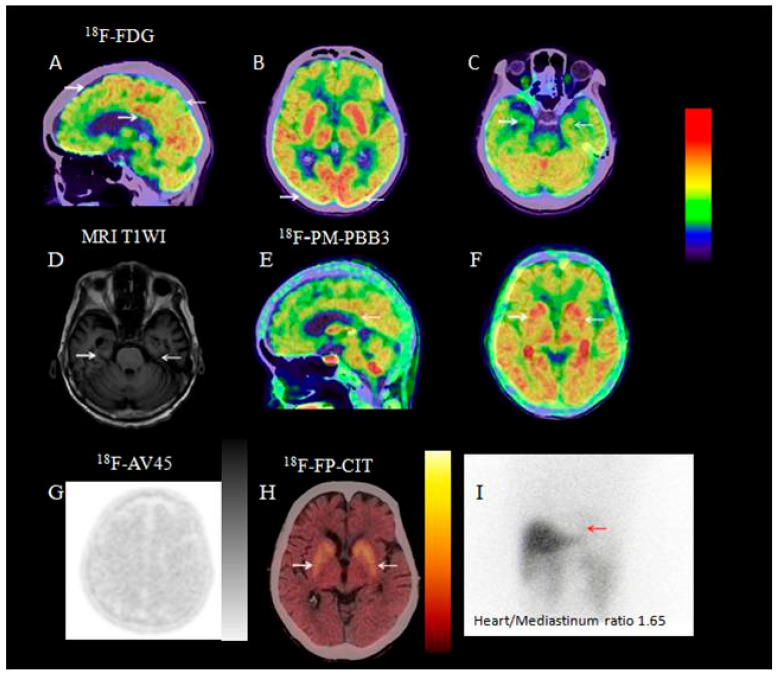
A 78-year-old female patient with 1-year bradykinesia, 6-month resting tremor, and 2-month neuropsychiatric symptoms. ^18^F-FDG PET/CT shows bilateral occipital and medial temporal hypometabolism, with preserved posterior cingulate metabolism ((**A**–**C**); white arrows). MRI T1WI shows bilateral medial temporal atrophy ((**D**); white arrow). ^18^F-PM-PBB3 PET shows tau deposition in the cerebral cortex and basal ganglia ((**E**,**F**); white arrows). ^18^F-AV45 PET shows cerebral Aβ deposition (**G**). ^18^F-FP-CIT PET/CT shows reduced bilateral basal ganglia dopamine transporter distribution, more severe on the right ((**H**); white arrow). ^131^I-MIBG scintigraphy shows a heart/mediastinum ratio of 1.65 ((**I**); red arrow), indicating cardiac sympathetic denervation. (**Note:** The images related to this case are adapted from Figure 1 in the author’s previously published literature [14]. In this study, the presentation order of the images has been adjusted as the patient’s case was included for analysis, while the core content of the images remains consistent with the original and has not been modified.).

Two patients in the DLB group underwent ^18^F-PM-PBB3 PET/CT, with 1 testing positive (Figure 8).

## 4. Discussion

Among neurodegenerative diseases, DLB has the second-highest incidence of neurodegenerative dementia after AD. However, the clinical diagnosis rate of DLB during life is lower than the post-mortem pathological confirmation rate [31,32]. Reasons for underdiagnosis include: (1) overlapping features with other neurological diseases [33]; (2) diagnostic difficulty based solely on clinical features; (3) potential comorbidity with AD [23]; (4) insufficient clinical awareness. Notably, drugs commonly used to treat other dementias (e.g., traditional antipsychotics) may exacerbate DLB symptoms [34], potentially inducing neuroleptic malignant syndrome and even endangering life [35]. Thus, accurate diagnosis is critical for developing appropriate treatment plans.

Core clinical features of DLB include fluctuating cognitive impairment, recurrent visual hallucinations, REM sleep behavior disorder, and parkinsonian symptoms. Parkinsonian symptoms include bradykinesia, resting tremor, or rigidity. According to guidelines [11], probable DLB is diagnosed with two or more core clinical features (with or without positive supportive biomarkers) or one core clinical feature with one or more positive supportive biomarkers. Vivid, recurrent visual hallucinations are characteristic of DLB, appearing early with high incidence, often involving complex, lifelike figures, animals, or scenes (e.g., seeing strangers in the room or small animals moving), with patients firmly believing in their reality and sometimes accompanied by delusions [36]. In contrast, AD patients rarely experience visual hallucinations, and PD patients may develop simple visual hallucinations (e.g., shadows, distorted objects) only in late stages (more than 10 years after motor symptom onset) [37]. Thus, visual hallucinations are a key differentiator between DLB and other neurodegenerative dementias [10].

In this study, all patients had parkinsonian symptoms and cognitive impairment; most (38/40, 95%) had vivid visual hallucinations, and 26 (65%) had REM sleep behavior disorder, consistent with probable DLB.

However, core clinical features of DLB overlap with those of other neurological diseases, causing diagnostic confusion. For example, fluctuating cognitive impairment resembles delirium; recurrent visual hallucinations may mimic schizophrenia; REM sleep behavior disorder also occurs in other neurodegenerative diseases (e.g., PD, MSA); and parkinsonian symptoms may lead to misdiagnosis as PD. Thus, DLB is difficult to diagnose based solely on clinical manifestations.

Regarding differentiation between PD and DLB, some scholars proposed the “1-year rule” [10]: cognitive decline developing more than 1 year after parkinsonian symptoms suggests Parkinson’s disease dementia (PDD), while cognitive decline preceding or occurring within 1 year of parkinsonian symptoms suggests DLB.

In our DLB group, over half (23/40, 57.5%) had initial parkinsonian symptoms, with an average interval of 24.43 months from parkinsonian symptoms to cognitive decline, inconsistent with the “1-year rule.” This indicates limitations of the “1-year rule” for distinguishing DLB from PDD based on symptom timing, suggesting a need for updates. Future development of early diagnostic methods based on biomarkers (e.g., α-Syn PET, cerebrospinal fluid α-Syn detection) may improve diagnostic accuracy [33]. Some scholars argue that DLB and PDD converge in clinical symptoms and pathology with disease progression, suggesting they may represent different stages of the same disease spectrum [38]. However, they are currently considered distinct entities.

Blood routine, biochemical tests, thyroid function, vitamin B12, and folate levels exclude systemic diseases causing cognitive impairment (e.g., anemia, liver/kidney dysfunction, electrolyte imbalance, hypothyroidism, vitamin B12/folate deficiency). Cerebrospinal fluid tests exclude infections or other neurological complications. Currently, no single specific biomarker confirms DLB.

Associations between DLB and Apo E genotype remain controversial, with studies suggesting potential influence of comorbid Aβ pathology. In our study, 4 of 8 patients with the Apo E3/4 genotype had positive Aβ PET/CT (indicating comorbid AD pathology), and 4 did not undergo Aβ PET/CT. We recommend Aβ PET/CT for DLB patients with the Apo E4 allele to assess cerebral Aβ deposition.

Structural imaging (MRI/CT) shows atrophy in the temporal, parietal, and occipital lobes in DLB, with relatively mild medial temporal atrophy—distinguishing it from AD, which typically shows severe medial temporal (e.g., hippocampal) atrophy.

Multimodal molecular imaging (PET) plays a crucial role in diagnosing neurodegenerative diseases including DLB.

No α-Syn tracer is currently available. Cerebrospinal fluid free α-Syn may be reduced in DLB patients [39], but its diagnostic value remains under investigation, and it is not specific for DLB [40].

^18^F-FDG PET/CT is the most widely used PET/CT modality. The typical metabolic pattern in DLB is occipital hypometabolism with relatively preserved posterior cingulate metabolism (posterior cingulate island sign) and relatively preserved hippocampal metabolism. In contrast, AD shows temporal and parietal hypometabolism with posterior cingulate hypometabolism. In our study, 75% of DLB patients had the typical FDG hypometabolic pattern, while 25% had atypical findings (generalized global hypometabolism or significant temporal/parietal hypometabolism) with indistinct posterior cingulate island sign, potentially indicating DLB complicated with AD (Figure 8). This explains why semi-quantitative analysis showed statistically significant posterior cingulate hypometabolism in the DLB group compared with controls.

Semi-quantitative methods (ROI and voxel-based analysis) precisely localize metabolic abnormalities. Our semi-quantitative analysis identified hypometabolism in the occipital visual cortex (responsible for visual processing) and inferior temporal gyrus (a key hub for converting “visual input” to “semantic understanding”), potentially explaining visual hallucinations in DLB.

Preserved hippocampal metabolism in DLB differentiates it from AD (characterized by hippocampal hypometabolism). Additionally, statistically significant increased amygdalar metabolism in DLB—consistent with its role as the “emotional center” and “survival hub”—may relate to anxiety symptoms via enhanced fear memory retention.

Thus, some DLB patients lack the typical ^18^F-FDG PET/CT pattern. Multimodal molecular imaging is recommended for clinically suspected cases with atypical FDG metabolism to clarify cerebral pathology.

Aβ PET/CT detects cerebral Aβ deposition, aiding early diagnosis of AD and other neurodegenerative diseases [41]. For example, it identifies abnormal cerebral Aβ in individuals with AD family history or mild cognitive impairment, enabling early intervention. The 2023 Expert Consensus on Amyloid PET in Alzheimer’s Disease [42] notes that Aβ PET/CT cannot differentiate AD from DLB, as some DLB patients have cerebral Aβ deposition. A study found that 61% of DLB patients met AD pathological criteria post-mortem [43]. However, Aβ PET/CT clarifies Aβ deposition in suspected DLB-AD comorbidity. We recommend Aβ PET/CT for probable DLB patients with severe cognitive impairment or ^18^F-FDG PET/CT showing occipital hypometabolism with posterior cingulate preservation plus global or temporal hypometabolism, as DLB-AD comorbidity is not rare. In our study, 9 of 12 such patients (81.8%) had positive Aβ PET/CT, indicating AD pathology. Two of these 9 underwent tau PET/CT, with 1 showing tau deposition in the temporal and parietal association areas—meeting the ATN criteria for AD, confirming DLB-AD comorbidity.

The high rate of positive Aβ PET/CT in our cohort aligns with international studies, indicating that positive Aβ PET/CT does not exclude DLB. Semi-quantitative CL values were ≤30 in 3 DLB patients, and the CL values of the other 2 patients were close to 30. combined with visual analysis, we hypothesize that Aβ deposition in the cerebellar cortex (increasing cerebellar SUV) may lower CL values (even to negative), suggesting visual analysis is critical for interpreting Aβ PET/CT in DLB, with semi-quantitative analysis alone insufficient. Furthermore, we propose that the mismatch between positive visual analysis and CL values ≤ 30 in some cases arises from the inherent design of the CL value calculation. Specifically, the CL value was developed to quantify the amount of cerebral amyloid deposition in AD patients: a CL value ≤ 30 indicates a low level of cerebral amyloid deposition in AD patients. However, directly applying this metric to DLB patients has limitations. Additionally, DLB patients with positive Aβ PET/CT had more cerebellar Aβ deposition than AD patients—an unstudied phenomenon warranting further research. Therefore, our future research aim is to enroll more DLB patients with positive Aβ deposition to establish a DLB-specific reference range for CL values. Before the semi-quantitative reference range for Aβ PET deposition in DLB patients is established, visual analysis should be the primary method for interpreting Aβ PET results in DLB patients.

Eleven patients in our cohort underwent ^18^F-FP-CIT PET/CT, with 10 showing reduced DAT uptake—reflecting substantia nigra dopamine system damage [44], consistent with previous studies. Contrary to guidelines suggesting symmetric DAT reduction in DLB and asymmetric reduction in PD, 5 of 10 DLB patients with reduced DAT uptake had asymmetric reduction—consistent with Lee YG’s report [44]. Notably, all asymmetric cases showed more severe right-sided reduction, whose significance remains unclear. Thus, reduced DAT uptake supports DLB diagnosis, but the value of symmetric/asymmetric distribution or laterality in differentiating PD from DLB requires larger studies. Since AD and frontotemporal dementia (FTD) typically lack reduced DAT uptake [45], DAT PET aids DLB differentiation from AD and FTD [46].

^131^I-MIBG reflects cardiac sympathetic innervation [47]. Autonomic dysfunction in DLB and PD reduces cardiac ^131^I-MIBG uptake [48]. All 15 patients in our study had cardiac sympathetic denervation (100% sensitivity). Thus, MIBG scintigraphy aids DLB differentiation from AD and FTD. However, other conditions reducing cardiac sympathetic innervation (e.g., heart failure, diabetic cardiac autonomic neuropathy) or certain drugs may cause false positives [49]. Drugs affecting ^131^I-MIBG uptake (e.g., tricyclic antidepressants, certain antihypertensives) should be discontinued before testing [50], and other causes of cardiac sympathetic denervation excluded to ensure accuracy.

## 5. Conclusions

In conclusion, DLB presents with early parkinsonian symptoms and cognitive impairment, with high incidences of visual hallucinations and sleep disorders. Imaging features—occipital hypometabolism and posterior cingulate island sign on ^18^F-FDG PET/CT, cardiac sympathetic denervation on ^131^I-MIBG scintigraphy, and dopaminergic neuron damage in the basal ganglia on ^18^F-FP-CIT PET/CT—aid DLB diagnosis. For patients with severe cognitive impairment, supplementary Aβ PET/CT detects potential Aβ pathology, indicating possible comorbidity with AD and explaining severe cognitive impairment.

## Figures and Tables

**Table 1:** General Clinical Data of the DLB Group and Control Group.

Parameter	DLB Group (*n* = 40)	Control Group (*n* = 40)
Sex		
Male	29	29
Female	11	11
Age (years)	72.1 ± 6.9	72.1 ± 8.0
MMSE score	18.1 ± 5.5	-
Clinical manifestations, *n* (%)		-
Cognitive impairment	40 (100%)	-
Parkinsonian symptoms	40 (100%)	-
Visual hallucinations	38 (95%)	-
REM sleep behavior disorder	26 (65%)	-

**Table 2:** Comparison of ^18^F-FDG SUVR between the DLB Group and Control Group Based on ROI Analysis.

Brain Region	^18^F-FDG SUVR		Statistical Values	
	DLB Group (*n* = 40)	Control Group (*n* = 40)	t-Value	*p*-Value
Calcarine_R	1.12 ± 0.13	1.23 ± 0.13	−3.69	0.00
Calcarine_L	1.12 ± 0.15	1.22 ± 0.15	−3.03	0.00
Cuneus_R	1.21 ± 0.14	1.34 ± 0.14	−4.18	0.00
Cuneus_L	1.34 ± 0.18	1.48 ± 0.19	−3.04	0.00
Occipital_Sup_R	0.95 ± 0.12	1.08 ± 0.11	−5.07	0.00
Occipital_Sup_L	1.20 ± 0.15	1.32 ± 0.16	−3.60	0.00
Occipital_Mid_R	0.89 ± 0.11	1.04 ± 0.10	−6.29	0.00
Occipital_Mid_L	0.95 ± 0.11	1.08 ± 0.12	−4.99	0.00
Occipital_Inf_R	1.05 ± 0.11	1.21 ± 0.10	−6.57	0.00
Occipital_Inf_L	1.08 ± 0.12	1.21 ± 0.12	−4.83	0.00
Precuneus_R	1.05 ± 0.10	1.21 ± 0.13	−6.43	0.00
Precuneus_L	1.14 ± 0.12	1.33 ± 0.14	−6.56	0.00
Angular_R	0.87 ± 0.09	1.04 ± 0.11	−7.32	0.00
Angular_L	0.81 ± 0.09	0.96 ± 0.11	−6.62	0.00
Temporal_Mid_R	0.91 ± 0.07	1.03 ± 0.09	−6.53	0.00
Temporal_Mid_L	0.89 ± 0.08	0.97 ± 0.08	−4.46	0.00
Temporal_Inf_R	0.86 ± 0.06	0.97 ± 0.06	−7.60	0.00
Temporal_Inf_L	0.89 ± 0.07	0.95 ± 0.06	−4.32	0.00
Hippocampus_R	0.89 ± 0.07	0.86 ± 0.06	1.72	0.09
Hippocampus_L	0.83 ± 0.07	0.81 ± 0.05	1.22	0.23
Cingulate_Post_R	0.62 ± 0.07	0.68 ± 0.10	−3.04	0.00
Cingulate_Post_L	0.73 ± 0.08	0.81 ± 0.14	−2.81	0.00
Amygdala_R	0.83 ± 0.08	0.79 ± 0.06	2.66	0.02
Amygdala_L	0.81 ± 0.10	0.77 ± 0.06	2.40	0.01

Note: Data are expressed as mean ± standard deviation. Abbreviations: ROI: region of interest; SUVR: standardized uptake value ratio.

**Table 3:** Clusters with Significant ^18^F-FDG PET Differences Between the DLB Group and Control Group at the Whole-Brain Voxel Level.

Brain Region of Cluster	Cluster Size (Voxels)	Peak t	Peak MNI Coordinates		
			x	y	z
DLB < HC					
Cluster 1	29,280	−11.5121	−44	−74	36
Occipital_Mid_L (aal)	2736				
Temporal_Mid_L (aal)	2409				
Parietal_Inf_L (aal)	1864				
Temporal_Mid_R (aal)	1836				
Precuneus_L (aal)	1653				
Precuneus_R (aal)	1609				
Temporal_Inf_L (aal)	1536				
Occipital_Mid_R (aal)	1460				
Angular_R (aal)	1460				
Temporal_Inf_R (aal)	1337				
Parietal_Sup_L (aal)	1167				
Angular_L (aal)	1104				
Parietal_Inf_R (aal)	1056				
SupraMarginal_R (aal)	847				
Parietal_Sup_R (aal)	814				
Occipital_Inf_L (aal)	701				
Occipital_Sup_L (aal)	669				
SupraMarginal_L (aal)	656				
Occipital_Sup_R (aal)	491				
Fusiform_L (aal)	479				
Cuneus_L (aal)	402				
Cuneus_R (aal)	279				
Occipital_Inf_R (aal)	234				
Temporal_Sup_R (aal)	216				
Calcarine_R (aal)	194				
Lingual_R (aal)	185				
Lingual_L (aal)	147				
Calcarine_L (aal)	136				
Temporal_Sup_L (aal)	128				
Cingulum_Post_L (aal)	70				
Cingulum_Post_R (aal)	33				
Cluster 2	1037	−6.4095	42	32	38
Frontal_Mid_R (aal)	908				
Frontal_Inf_Tri_R (aal)	57				
Cluster 3	2379	−6.5266	−32	24	52
Frontal_Mid_L (aal)	1628				
Frontal_Inf_Tri_L (aal)	473				
Frontal_Sup_L (aal)	104				

Note: MNI: Montreal Neurological Institute; DLB: Dementia with Lewy bodies; HC: healthy control.

**Table 4:** Semi-Quantitative Analysis Results of ^18^F-AV45 PET/CT.

	CL Value	Cerebral SUV (g/mL)	Cerebellar SUV (g/mL)
Case			
1	−17	0.55	0.54
2	54	0.99	0.71
3	18	0.68	0.57
4	−34	0.68	0.73
5	−50	0.25	0.30
6	31	0.67	0.53
7	31	1.04	0.81
8	53	0.99	0.71
9	42	1.04	0.77

Note: CL: Centiloid; SUV: standardized uptake value.

## Data Availability

The original contributions presented in this study are included in the article. Further inquiries can be directed to the corresponding author.
